# Bloodstream infections and Blood-Brain barrier Permeability: An observational cohort study

**DOI:** 10.1016/j.bbi.2026.106518

**Published:** 2026-07

**Authors:** Jonathan Underwood, Kate Davies, Sam Loveless, Mara Cercignani, Nicholas G. Dowell, Eva Periche-Tomas, Itamar Ronen, John Evans, James E. McLaren, Neil A. Harrison

**Affiliations:** a Division of Infection and Immunity, Cardiff University, Cardiff, UK; b Department of Infectious Diseases, Cardiff and Vale University Health Board, Cardiff, UK; c Systems Immunity Research Institute, Cardiff University, Cardiff, UK; d Division of Psychological Medicine and Clinical Neurosciences, Cardiff University, Cardiff, UK; e Cardiff University Brain Research Imaging Centre (CUBRIC), Cardiff University, Cardiff, UK; f Department of Psychiatry, University of Oxford, Oxford, UK; g Clinical Imaging Sciences Centre, Brighton and Sussex Medical School, University of Sussex, Falmer, Brighton, UK

**Keywords:** Bloodstream infections, Blood-brain barrier, Cognitive dysfunction, Neuroimaging, DCE-MRI

## Abstract

**Background:**

Sepsis associated encephalopathy is a poorly understood yet common complication of sepsis. Immune challenge and animal models implicate blood–brain barrier (BBB) dysfunction and microglial activation in the hippocampus as key drivers of pathology. In this exploratory study, we used bloodstream infections (BSI) as a model of severe infection to investigate the relationship between changes in cognition with plasma biomarkers of brain injury, BBB permeability and microglial activation quantified using advanced neuroimaging.

**Methods:**

In this longitudinal exploratory study we recruited patients hospitalised with BSI as well as age matched controls. Cognition was assessed at the bedside and at convalescense using a CANTAB battery. MRI scanning was performed as soon as practicable after hospital discharge. Hippocampal and thalamic BBB permeability was assessed using dynamic enhanced contrast MRI (DCE-MRI). Microglial morphometry was assessed using diffusion-weighted magnetic resonance spectroscopy (DW-MRS) of the left thalamus. Plasma glial fibrillary acidic protein (GFAP) and neurofilament light protein (NFL) were quantified using Single molecule array (SiMoA) technology.

**Results:**

21 patients with BSI and 16 age matched controls were enrolled (median [IQR] 64.3 [51.1–68.5] years). Patients with BSI had poorer cognitive function, particularly attention (RVP A-prime z-score −1.08 [95% CI −1.75, −0.61], p < 0.001). This deficit correlated with the magnitude of the inflammatory response quantified by peak c-reactive protein (CRP). Furthermore, patients with BSI had higher concentrations of NFL, which was correlated with poorer cognitive function and more severe illness. At the scanning visit (median: 32 days from discharge) cognition had largely normalised and was similar to controls. However, patients with BSI had significantly more depressive symptoms (p < 0.01). There were no differences in BBB permeability or metabolite apparent diffusion coefficients between patients with BSI and controls.

**Conclusions:**

Our findings suggest that BSI are associated with acute, inflammation-mediated brain injury. However, cognitive dysfunction, particularly in the domain of attention, is temporary and not associated with changes in BBB permeability or microglial activation at convalescence. Given persistent mood dysfunction and elevated biomarkers of neuronal injury longitudinal study is warranted to determine long-term sequelae of BSI and other severe infections.

## Highlights

• Bloodstream infection provides a novel model of severe systemic inflammation.
• Bloodstream infection causes acute, inflammation-associated deficits in attention.
• Elevations in biomarkers of brain injury correlate with severity of illness.
• At convalescence cognition normalised, but depressive symptoms persisted.
• No evidence of persistent blood–brain barrier dysfunction or microglial activation.

## 1 Introduction

Bloodstream infections (BSI) are common life-threatening diseases that cause sepsis with a 30-day mortality in excess of 15% (Underwood et al., 2024). In those who survive, the morbidity is significant with short term cognitive impairment (known as delirium or sepsis associated encephalopathy − SAE) frequently observed by clinicians, patients and their families. SAE is acute neurological dysfunction associated with systemic or peripheral infection and a dysregulated host immune response without overt central nervous system infection. It is a frequent complication of sepsis, affecting up to 70% (Carter and Underwood, 2022, Gofton and Young, 2012). However, despite its ubiquity it is poorly understood in part because of the overlap with physiological sickness behaviours.

SAE is a heterogenous condition due to variability in culprit pathogens, pathophysiology and treatment. As such, the relationship between SAE and long-term cognitive impairment is unclear. However, there are data to suggest that significant infections (i.e. that cause hospitalisation) are associated with subsequent significant cognitive decline (Shah et al., 2013). A similar but weaker relationship with incident dementia was observed with milder infections using linked population data from the UK with the largest hazard ratio reported for patients with more severe infections and sepsis (Muzambi et al., 2021). Furthermore, Iwashyna *et al* (Iwashyna et al., 2010) reported an absolute increase in the prevalence of severe persistent cognitive impairment (i.e. dementia) from 6.1% pre-sepsis to 16.7% in survivors of severe sepsis three years later. Similarly, systemic inflammatory events, the majority of which were secondary to infections, have been reported to accelerate the rate of cognitive decline in patients with established Alzheimer’s disease (Holmes et al., 2009) mirroring experimental murine models of tauopathy (Torvell et al., 2019). Together, these data suggest that systemic inflammation precipitated by infection is associated with the hastening of the onset of dementia and accelerated progression in those with an established diagnosis.

Putative pathophysiological methods of SAE include blood–brain barrier (BBB) dysfunction, endothelial injury, micro- and macro-circulatory dysfunction, microglial activation, neuronal injury and death (Carter and Underwood, 2022, Gofton and Young, 2012, Widmann and Heneka, 2014). The BBB plays a central role in maintaining the microenvironment of the central nervous system − protecting it from systemic insults including infection. It is a dynamic, multicellular interface formed by specialised brain endothelial cells interconnected by tight junctions, supported by pericytes, astrocytic end feet, and the extracellular matrix. BBB ‘permeability’ reflects multiple, partially independent processes, including paracellular tight-junction integrity, transcellular transport, vesicular trafficking, endothelial activation and immune cell transmigration. BBB dysfunction has been identified as a key pathophysiological mechanism of SAE, with persistent dysfunction implicated in progressive cerebral injury, however *in vivo* data in humans are limited (Nwafor et al., 2019, Varatharaj and Galea, 2017). BBB dysfunction is found early in many neurodegenerative conditions such as AD and multiple sclerosis and can be assessed *in vivo* using dynamic contrast-enhanced magnetic resonance imaging, DCE-MRI (Cramer et al., 2014, Varatharaj and Galea, 2017).

DCE-MRI provides a quantitative, *in vivo* measure of the transfer of gadolinium-based contrast agents from the intravascular compartment into brain tissue, typically parameterised as the volume transfer constant (k_trans_). Under most modelling assumptions k_trans_ reflects macroscopic paracellular permeability and/or capillary surface area and is therefore sensitive to overt leakage of small solutes across the endothelial barrier. As such, k_trans_ can be used as a proxy for this aspect of BBB permeability. A rat model of sepsis, involving injection of lipopolysaccharide (LPS – a key component of Gram-negative bacteria’s cell membrane), reported short- and long-term BBB dysfunction measured using dynamic DCE-MRI (Towner et al., 2018). These effects were particularly marked in the hippocampus and cerebral cortex. Hippocampal atrophy is recognised as one of the hallmarks of AD. Brain injury and BBB dysfunction in this region has been reported in a murine caecal-ligation and puncture model of SAE (Bozza et al., 2009), an experimental model of systemic inflammation in humans (Harrison et al., 2014) as well in normal ageing and greatest in those at genetic risk of Alzheimer’s disease (Montagne et al., 2020, 2015). Taken together, these findings suggest that this region of the brain is particularly vulnerable to injury associated with systemic inflammation associated with infection. Novel techniques such as diffusion-weighted MR spectroscopy (DW-MRS) can give further insights into the pathophysiology of SAE. Recent data from a LPS human immune challenge model, showed this technique was sensitive to subtle changes in glial morphometry induced by systemic inflammation (Marco et al., 2022). Furthermore, this technique has also been shown to be sensitive to microglial reactivity to even very low levels of gut-derived endogenous LPS concentrations (Birg et al., 2024).

The heterogeneous aetiology of sepsis and SAE are acknowledged as one of the biggest challenges to its understanding. Pre-existing brain injury and its associated microglial activation and BBB dysfunction is likely to be a major risk factor for developing SAE and persistent cognitive impairment following severe infection. It is unclear if SAE is exaggerated sickness behaviour in a vulnerable brain and whether this fully recovers or leads to acute and chronic brain injury. In this exploratory study, we used BSI as a model of sepsis, but with an unambiguous bacterial pathogen, to identity potential mechanisms underpinning the relationship between severe infection and persistent cognitive decline. We hypothesised that BSI would be associated with acute and chronic cognitive impairment mediated by increased hippocampal BBB permeability.

## 2 Methods

### 2.1 Participants

The study design is summarised in Supplementary Fig. 1. Hospitalised adults, aged over 18, with *Staphylococcus aureus* and *Escherichia coli* BSI were prospectively enrolled in an observational cohort study at the University Hospital of Wales, Cardiff, UK. Demographically comparable controls, who had not been hospitalised with BSI or COVID-19 in the preceding year, were recruited from friends and relatives of patients and volunteers who responded to electronic or emailed advertisements around the Cardiff area. Participants were excluded if pregnant, could not have MRI scanning, or considered too unwell/limited life expectancy (at the discretion of the investigator). Fully informed, written consent was collected in all participants. The study protocol was approved by the Health Research Authority and Research Ethics Committee (REC ref: 21/WA/0202; IRAS project ID: 277598). A total sample size of 32 was based on an increase of 25% in hippocampal BBB permeability in people with mild cognitive impairment compared to controls, assuming a mean ± SD Ktrans 1.2 ± 0.3 x10-3 min^−1^ in controls (Montagne et al., 2015) with 80% power and α of 0.05.

### 2.2 Cognitive function testing and assessment of mood

Patients with BSI had testing at the bedside using the 4AT (Bellelli et al., 2014) and using a computerised CANTAB battery testing: choice reaction time (RTI), motor speed (MOT), attention (rapid visual information processing – RVP), working memory (spatial span – SSP) and executive function (spatial working memory – SWM). These tests were chosen as they were deliverable at the bedside, had minimal practice effects allowing repeated testing as well as testing major cognitive domains likely to be affected by SAE. Additionally, participants completed the hospital anxiety depression scale (HADS). At the scanning visit all participants (BSI and controls) completed the same CANTAB battery and HADS. Additionally, all participants completed the mini-Addenbrooke's Cognitive Examination (mini-ACE) and the four mountains test, a sensitive test of allocentric spatial memory to assess hippocampal function (Chan et al., 2016, Hartley et al., 2007). PHQ-9 and GAD-7 were also collected at this point for all participants to assess mood. These additional tests were not administered to the BSI group during their inpatient stay due to their difficulty and the limited time it is ethical and practical to administer cognitive tests in hospitalised patients with infections.

### 2.3 Brain injury biomarkers

Glial fibrillary acidic protein (GFAP) and neurofilament light protein (NFL) were quantified from plasma using Single molecule array (SiMoA) technology. We used an HD-X analyser, a fully automated multiplex digital immunoassay instrument providing ultra-sensitive measurements over a wide dynamic range and with low coefficients of variance. We used Quanterix Neurology 4-plex A array kits (cat# 102153), array discs (cat#103347) and assay buffers (cat#100488), following the manufacturer’s protocol.

### 2.4 MRI acquisition and analysis

Participants attended for MRI scanning at the Cardiff University Brain Research Imaging Centre (CUBRIC) as soon as practicable after discharge (BSI group) aiming to be within 30-days. MRI data was acquired using a Siemens 3 T Prisma.

#### 2.4.1 Structural MRI

High-resolution T1-weighted structural MRI scans were acquired using a magnetization-prepared rapid gradient echo sequence (TR = 2,100 ms; TE = 3.24 ms; TI = 850 ms, flip angle = 8 deg, FOV 256 × 256 mm^2^, 176 sagittal slices, resulting in 1 mm isotropic voxels). This was processed using FreeSurfer version 7.1.1 (https://surfer.nmr.mgh.harvard.edu), employing the standard automated ‘recon-all’ pipeline (Fischl, 2012). This includes skull stripping, intensity normalisation, automated tissue classification, reconstruction of the white and pial cortical surfaces, and segmentation of subcortical structures using a probabilistic atlas–based approach. For global cortical morphometry, mean cortical thickness was extracted separately for each hemisphere for primary analyses. Selected bilateral subcortical structures (hippocampus and thalamus) and global volumetric measures, including total cortical grey matter volume, were obtained from the automated segmentation. All segmentations underwent visual quality control and no scans were excluded due to segmentation failure. Due to the known associations with cortical thickness and volumetric measures age, sex (cortical thickness) as well as estimated intracranial volume (ICV) (volumetric measures) were used to adjust structural measures in linear regression analyses. Given the exploratory and descriptive aim of assessing broad structural similarity rather than regional localisation, analyses focused on global summary measures rather than vertex-wise or voxel-wise comparisons.

#### 2.4.2 Dynamic contrast enhanced MRI (DCE-MRI)

Pre-contrast T1 maps were acquired based on the variable flip angle approach (Venkatesan et al., 1998) that involves collecting spoiled gradient echo acquisitions at flip angles 6, 30 and 33 degrees corrected for B1 inhomogeneities.

DCE images were collected using a spoiled gradient echo pulse sequence (TR = 3 ms; TE = 1.37 ms; flip angle 9 deg; FOV = 240 x 240 x 180 mm; matrix size = 96 x 68 x 50, reconstructed to 96 x 68 x 72; time resolution = 7 s; number of volumes = 150; total scan time 17.5 min). Standard dose contrast agent (dotarem, gadoteric acid; 0.1 mmol/kg) was administered after collecting 12 volumes of pre-contrast baseline data. Images were processed using a custom pipeline using FSL (v.6.0.1). Briefly, individual DCE volumes were cropped and motion corrected using MCFLIRT (Jenkinson et al., 2002) before co-registration with T1 map and high resolution T1 structural image using FLIRT (Klein et al., 2009). DCE data was denoised with a custom filter and k_trans_ maps were generated by modelling the DCE signal using the Fabber Bayesian forward model fitting framework to implement an extended one-compartment Tofts model (Chappell et al., 2009, Groves et al., 2009). The superior sagittal sinus was used to determine the vascular input function. In reality, a gadolinium concentration profile measured in a venous ROI is an ”output” function. However, the larger cross-sectional area of this vein provides better signal-to-noise and contrast-to-noise ratio, is less sensitive to motion and has been shown to have a similar concentration profile to that in the artery (Sourbron et al., 2009, Thrippleton et al., 2019). Blood plasma fraction was determined from haematocrit values measured at screening for controls or the day of scanning for patients. Model fit was assessed using root-mean squared error and voxels greater than 10% were excluded from further analysis. T1 data was segmented using FIRST and FAST (Patenaude et al., 2011) to generate region of interest masks (grey matter, white matter, thalamus and hippocampus) which were used to determine the median K_trans_ for each region which were used in subsequent analyses. An example of modelled Ktrans data is shown in Supplementary Fig. 2.

#### 2.4.3 MRS and DW-MRS

A single 4.5 cm^3^ volume of interest (VOIs) was placed in the left thalamus, using a T1-weighted MPRAGE as anatomical reference. VAriable Power and Optimized Relaxations delays (VAPOR) suppressed (Tkáć and Gruetter, 2004) and unsuppressed MRS data was collected using a STimulated Echo Acquisition Mode (STEAM) sequence: TE 10 ms; TR 3 s; TM 43 ms; 64 averages (water suppressed).

Spectra were processed and quantified as previous described using the totally automatic robust quantitation in nuclear MR (TARQUIN) algorithm (Scott et al., 2016, Wilson et al., 2011). Briefly, the absolute concentrations of the following metabolites were quantified after eddy-current correction, using an unsuppressed water reference signal: choline (Cho), creatine (Cr), glutamate and glutamine (Glx), myo-inositol (MI) and N-acetyl-aspartate (NAA). All spectra were visually inspected to assess model fit and poor fits were rejected. Additionally, spectra were excluded if the signal-to-noise-ratio was < 4 or the metabolite full-width half-maximum was > 0.1 ppm.

DW-MRS was collected using a semi-Localization by Adiabatic SElective Refocusings sequence (semi-LASER) with a bipolar gradient scheme (Genovese et al., 2021) in the same left thalamus voxel. Acquisition parameters matched those described by De Marco et al (Marco et al., 2022) and included a TE of 100 ms, a TR of 5 s, a spectral width of 2500 kHz, and 1024 complex data points. Diffusion weighting was applied using one measurement at b = 0 s/mm^2^ and three at b = 3823 s/mm^2^, with diffusion gradients oriented along three orthogonal directions. Each diffusion condition was acquired with 32 signal averages (NSA = 32). An additional short scan (NSA = 4) without water suppression was conducted to enable eddy current correction. B_0_ shimming was optimized using the FAST(EST)MAP method (Gruetter and Tkáč, 2000), which employs echo-planar signal trains and projection-based mapping.

Data were processed and analysed using a custom MATLAB pipeline as previously described (Marco et al., 2022). Briefly, weighted average free induction decay (FID) data were generated based on the signal detected in each receive coil. Eddy current correction was performed using the water data acquired in the same conditions of the DWS data. Spectra were phase corrected based on the NAA peak and included in further averaging and analysis only if the peak amplitude was greater than the NAA peak minus 4x standard deviation of noise estimated from the spectra in a region without spectral contribution. Spectra were quantified using linear prediction singular value decomposition (LPSVD). Average diffusion coefficient (ADC) for each metabolite (Cho, Cr and NAA) were calculated from the average spectra acquired each orthogonal diffusion gradient at high b*-*value.

### 2.5 Statistical analyses

Group demographic and clinical characteristics were compared with non-parametric tests as appropriate. Cognitive scores were demographically corrected to z-scores using CANTAB population norms where data was available (adjusted for age, sex and level of education). If not available raw scores were compared. Longitudinal comparisons of measures of cognitive function and brain injury biomarkers were analysed using longitudinal mixed-effects models using with lme4 package in R (Bates et al., 2015). For longitudinal analysis study visit was a fixed effect (i.e. baseline and scanning) with a random effect for each participant for each outcome of interest. Correlations between neuroimaging, cognitive and biomarker data were determined using Spearman’s rank correlation coefficient. Analyses were performed using *R* version 4.4.2.

## 3 Results

### 3.1 Participants

21 patients with BSI (9 *Staphylococcus aureus* and 12 *Escherichia coli,* none with central nervous system infections) and 16 age matched controls were enrolled (see Table 1 for further demographic details). 13 (62%) patients met criteria for sepsis based on their admission Sequential Organ Failure Assessment (SOFA) score. Five patients with BSI withdrew or were lost to follow-up before their scanning visit. The median (IQR) time from hospital discharge to scanning visit was 32 (20–62) days.

**Table 1:** Demographics.

**Characteristic**	**Control** N = 16^a^	**BSI** N = 21^a^	**p-value**^b^
**Age (years)**	64 (60 – 68)	66 (51 – 72)	0.60
**Sex**			0.019
Female	7 (44)	17 (81)	
Male	9 (56)	4 (19)	
**Ethnicity**			>0.99
Black	0 (0)	0 (0)	
Asian	0 (0)	1 (4.8)	
White	16 (100)	20 (95)	
**Educational attainment**			0.11
Below US	4 (25)	10 (48)	
US	3 (19)	7 (33)	
Batchelor's	4 (25)	3 (14)	
PhD/higher	5 (31)	1 (4.8)	
**Scanning visit CRP (mg/L)**	1.0 (0.0, 1.5)	2.0 (1.5, 6.5)	0.018
Missing	0	5	
**Admission SOFA score**		2.00 (0.00 – 3.00)	
Missing		0	
**4AT score**			>0.99
0		16 (76)	
4		4 (19)	
5		1 (4.8)	
**Length of stay (days)**		10.0 (6.0 – 19.0)	
**Admission WCC x10^9**		14 (11 – 18)	
**Admission ANC x10^9**		12 (9 – 15)	
**Admission ALC x10^9**		0.65 (0.50 – 0.85)	
**Admission CRP (mg/L)**		248 (131 – 308)	
**Peak ANC x10^9**		10 (7 – 15)	
**Peak ALC x10^9**		0.60 (0.50 – 0.80)	
**Maximum CRP (mg/L)**		308 (235 – 370)	

WCC: white cell count. ANC: absolute neutrophil count. ALC: absolute lymphocyte count.

a Median (IQR); n (%).

b Wilcoxon rank sum test; Pearson's Chi-squared test; Fisher's exact test.

### 3.2 Cognitive function and mood

**Fig. 1:**
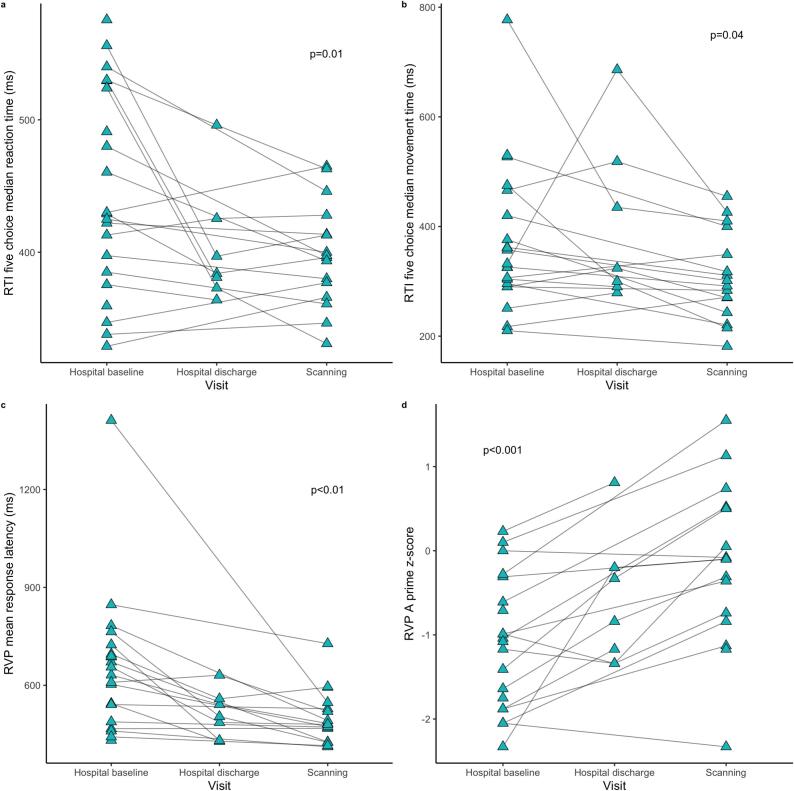
Longitudinal improvements in attention and processing speed for patients with bloodstream infection. Longitudinal changes in cognitive function of patients with bloodstream infection over time showing improvements in choice reaction time task (RTI) – panels a and b; and attention (rapid visual information processing task – RVP) – panels c and d. P-values calculated from longitudinal mixed-effects model of change from hospital baseline and scanning visit.

Despite the majority of patients with BSI not displaying overt delirium (16/21 [76%] had 4AT of 0) significant cognitive impaired was evident at hospital baseline on more detailed testing, with the greatest deficit observed in attention (RVP A-prime z-score −1.08 [95% CI −1.75, −0.61], p < 0.001). This measures a participant’s ability to discriminate target sequences from non-targets, independent of response bias, adjusted for age, sex and education. These deficits were most marked in patients with sepsis (RVP A-prime z-score median −1.41 vs. −0.51; p = 0.03, Supplementary Fig. 3). However, these deficits had largely normalised by the time of the scanning visit (median time: 32 days from hospital discharge, Fig. 1).

**Fig. 2:**
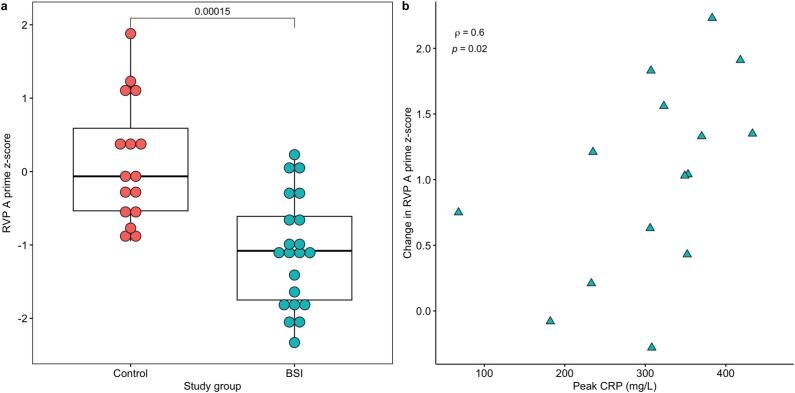
Acute impairment in attention is correlated with systemic inflammation. a: Patients with BSI have significant impairment in attention at study baseline (rapid visual information processing – RVP A-prime z-score). b: correlation between systemic inflammation measured by peak CRP concentration and change in attention between hospital baseline and follow-up. In contrast, measures of depression remained significantly elevated (Fig. 3). Depression and anxiety measures were not associated with acute illness severity or systemic inflammation (Supplementary Fig. 3).

**Fig. 3:**
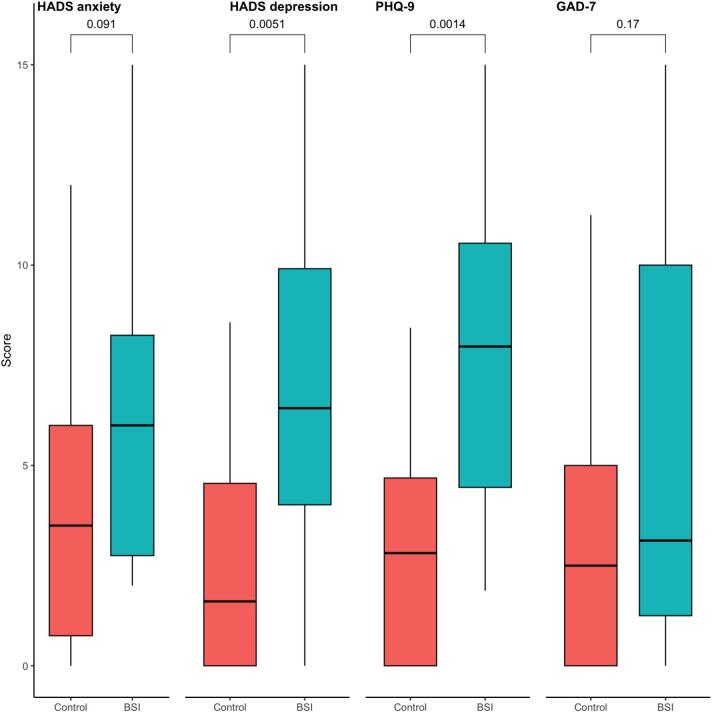
Mood dysfunction persists following bloodstream infection. Patients with BSI have significantly increased symptoms of depression and anxiety in convalescence compared with controls at scanning visit. HADS: Hospital anxiety and depression score. PHQ-9: Patient Health Questionnaire 9. GAD-7: General Anxiety Disorder 7.

Peak CRP, a proxy for the magnitude of the systemic inflammatory response, was negatively correlated with change in RVP A-prime z-score between hospital baseline and scanning visits (Fig. 2). At the scanning visit there was a trend for poorer performance in the four mountains test for patients with BSI compared to controls (median score 8.5 vs 11.5 respectively, p = 0.1) but no difference in mini-ACE scores (median score 28 vs 29 respectively, p = 0.2) Fig. 3.

### 3.3 Brain injury biomarkers

**Fig. 4:**
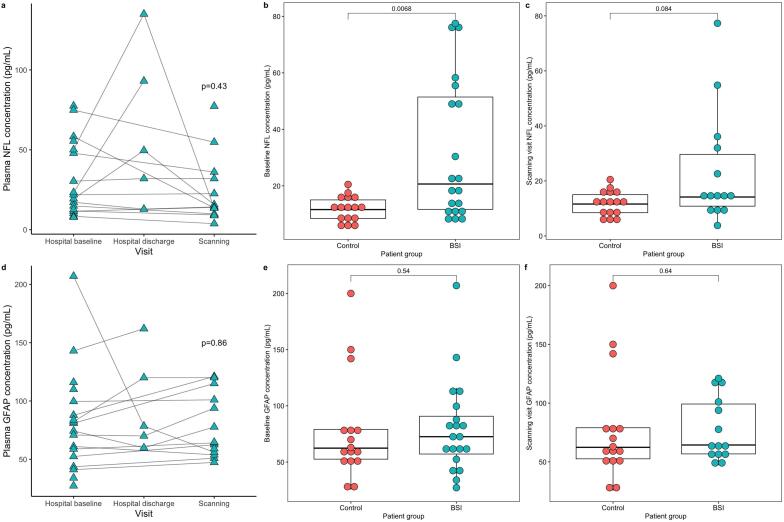
Axonal injury biomarker NFL is acutely elevated in patients with bloodstream infection. Plasma NFL but not GFAP is more elevated in patients with bloodstream infection at hospital baseline than at convalescence compared to controls. P values in panels a & d from longitudinal mixed-effects model comparing hospital baseline and scanning visit biomarkers. NFL: neurofilament light protein. GFAP: glial fibrillary acidic protein.

**Fig. 5:**
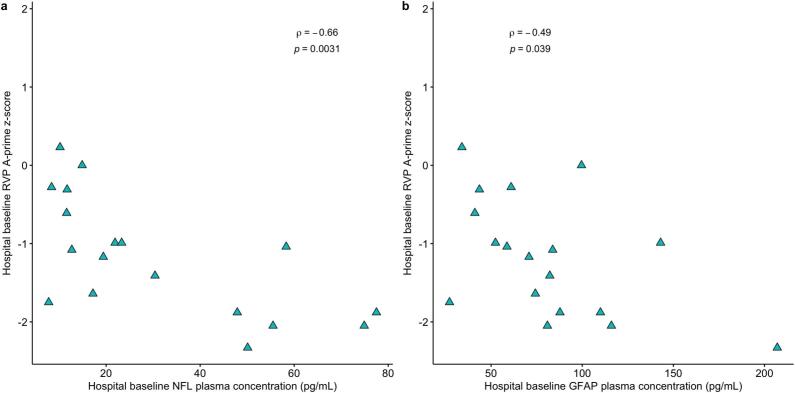
Higher concentrations of NFL and GFAP are associated with poorer attention at hospital baseline in patients with BSI. Scatterplots showing negative correlation between plasma NFL and GFAP and attention (quantified with rapid visual information processing – RVP A-prime z-score) – the cognitive domain most impaired in patients with bloodstream infection. NFL: neurofilament light protein. GFAP: glial fibrillary acidic protein.

Plasma NFL, but not GFAP, was elevated in patients with BSI, particularly at hospital baseline (Fig. 4). Furthermore, NFL strongly correlated with severity of acute illness – quantified by SOFA (Supplementary Fig. 4). In patients with BSI, both NFL and GFAP measured at hospital baseline were negatively correlated with initial RVP A-prime z-score (Fig. 5). Patients with BSI meeting sepsis criteria had higher concentrations of NFL (median 11.7 vs 47.9 pg/mL, p = 0.02, Fig. 5). There was a negative trend between hospital baseline NFL and RVP A-prime z-score at scanning visit (rho = -0.48, p = 0.08). However, there was no association between hospital baseline GFAP and RVP A-prime z-score at scanning visit (rho = -0.31, p = 0.27). Across the entire cohort, scanning visit NFL was negatively correlated with multiple measures of cognitive function (Supplementary Fig. 5).

### 3.4 Neuroimaging measures

Mean cortical thickness, total cortical grey matter volume, hippocampal and thalamic volumes did not differ between did not differ between patients with BSI and controls (p > 0.1 for all, supplementary figure 7). There were no differences in grey matter, white matter, hippocampal or thalamic K_trans_ between patients with BSI and controls. However, across the entire cohort, grey matter, hippocampal and thalamic K_trans_ negatively correlated with multiple measures of cognitive function (Supplementary Fig. 8). Furthermore, plasma NFL was associated with higher grey matter, hippocampal and thalamic K_trans_ (Supplementary Fig. 9) indicating these neuroimaging measures were sensitive to clinically significant neuropathology. Similarly, there were no differences in absolute metabolite concentrations nor metabolite ADC on DW-MRS between patients and controls (Supplementary Fig. 10). Plasma NFL and GFAP were not associated with absolute metabolite concentration nor metabolite ADC.

## 4 Discussion

In this cohort study, we demonstrate that BSI is associated with acute changes in cognition, particularly in attention, even in the absence of overt delirium. The magnitude of the deficit was correlated with peak CRP concentration and was more pronounced in patients with sepsis, implicating systemic inflammation as a key mediator. Furthermore, we observed acute elevations of NFL, a biomarker of axonal injury, with greater increases in those with more severe acute illness and poorer attention. Whilst cognition largely normalised a month after BSI mood dysfunction persisted. Notably, we found no evidence of persisting BBB dysfunction or microglial activation. Together, these findings imply that brain injury associated with BSI is largely transient. These findings offer some reassurance given the ubiquity of BSIs, yet underscore the need for longitudinal studies to assess long-term outcomes, particularly in light of persistent mood dysfunction and prior observational data linking sepsis to accelerated cognitive decline (Iwashyna et al., 2010, Muzambi et al., 2021).

To our knowledge, this is the first multi-modal study to evaluate acute brain injury following BSI. Contrary to our hypothesis, persistent hippocampal dysfunction and BBB disruption were not observed. While hospitalisation itself may impair cognition through sleep disruption, biomarker evidence of neuronal injury, most marked in severe illness and those with poorer cognitive function, suggests the transient cognitive impairment is not just sickness behaviour. Crucially, the correlation between attention deficits with the magnitude of the inflammatory response, with the greatest deficits observed in patients with sepsis, strongly implicates systemic inflammation as a driver of acute encephalopathy. Further study in larger cohorts is needed to disentangle which aspects of hospitalisation with BSI are associated with acute brain injury and how treatment affects this including an additional control group of patients hospitalised with non-inflammatory conditions. Pro-inflammatory cytokines such as IL-6 facilitate immune-brain communication and microglial activation (Dantzer et al., 2008, Sparkman et al., 2006). This pathway is corroborated by experimental models showing transient cognitive and mood impairments after immune challenges (Harrison et al., 2014, Marco et al., 2022, Periche-Tomas et al., 2025, Sandiego et al., 2015). Importantly, such models induce milder inflammation than seen in sepsis or our cohort. Taken together, we propose that infection-associated encephalopathy should be thought of as a continuum, with SAE representing its severe end. The interaction between inflammatory intensity and pre-existing neuropathology likely shapes clinical manifestations. However, confirming this hypothesis with immune challenge in vulnerable populations with existing neuropathology may be ethically problematic.

Consistent with immune challenge models reporting temporary effects on cognition, we found no convincing evidence of persistent cognitive impairment, increased BBB permeability nor microglial activation. This contrasts with recent data describing increased BBB permeability in patients with long-COVID associated brain fog (Greene et al., 2024). However, significant differences in patient population, methodology and time from infection to DCE-MRI (median 211 days for long-COVID vs 35 days here) and reduced brain volumes in the long-COVID patient groups likely explain the dissimilarities with our results. Given higher NFL and poorer cognitive function in patients with BSI acutely, and many studies reporting that NFL predicts future cognitive decline, longitudinal studies in larger cohorts are going to be crucial to determine long-term neurodegenerative trajectories.

Mood dysfunction following BSI is unsurprising and the causes are likely multifactorial. Psychological sequelae of critical illness plays an important role. However, inflammation has been linked to altered tetrahydrobiopterin (BH4) monoamine synthesis pathway (Vancassel et al., 2022) and tryptophan metabolism by microglial activation of indoleamine 2,3-dioxygenase (IDO) (Dantzer et al., 2008). Furthermore, chronic inflammatory conditions, e.g. rheumatoid arthritis, link inflammation to depression (Brock et al., 2023). The most compelling evidence of inflammation being causally associated with mood dysfunction comes from immune challenge models, where IL-6 again is the key cytokine of immune-brain communication. (Harrison et al., 2009, Wright et al., 2005). Although we did not measure IL-6 directly, markedly elevated CRP concentrations, a downstream marker of IL-6 activity, measured in patients with BSI, suggest it is highly likely that IL-6 concentrations were also very elevated (Ali et al., 2023). Murine studies reporting delayed mood recovery after immune challenge (Dantzer et al., 2008) align with our findings, warranting longitudinal assessment of mood trajectories.

Delirium, characterised by inattention, is a common complication of infection, yet standard bedside tools such as the 4AT may underestimate encephalopathy. Only a quarter of patients in our study met 4AT delirium criteria, yet they scored on average one standard deviation below population norms in the RVP task, a sensitive measure of sustained attention. This highlights the utility of objective cognitive testing to identify subtle deficits for clinical trials targeting infection-associated encephalopathy (e.g. with anti-inflammatories). Given the prevalence of BSIs and other severe infections, understanding long-term outcomes is critical. Modulation of the immune response in the acute phase may affect chronic trajectories of neurodegeneration. For example, minocycline, a licenced antibiotic, attenuates microglial activation and has protective effects in murine models of LPS induced cognitive impairment (Hou et al., 2016). However, clinical trials for other indications have been mixed.

## 5 Limitations

Studying acutely unwell patients in hospital is challenging. As such, our study has several limitations. MRI timing relative to acute illness precluded assessment of acute BBB or glial changes. Scans at convalescence allowed us to determine if there was persisting brain injury. However, as our research scanning facility is not on the acute hospital site it was not safe to transfer patients in the acute phase. Further, despite adequate power for detecting significant increases in hippocampal BBB permeability, our sample size limited sensitivity to detect subtle neuroimaging abnormalities and perform adjusted analyses or sex-specific analyses. As an exploratory study we also did not test for multiple comparisons. As such, findings should be interpreted as hypothesis-generating.

Importantly, DCE-MRI does not directly measure tight-junction protein expression, endothelial inflammatory signalling, transporter dysfunction, or leukocyte trafficking, nor is it sensitive to subtle or transient alterations in BBB function that do not permit gadolinium extravasation. As such, the normal DCE-MRI permeability metrics we report do not fully exclude the presence of BBB dysfunction. However, the neuroimaging techniques we used, particularly DW-MRS, are highly sensitive to neuroinflammation from immune challenges (Marco et al., 2022, Periche-Tomas et al., 2025) suggesting any persisting abnormalities are likely to be minimal.

Cognitive testing in acutely unwell patients in hospital potentially introduces variability due to environmental distractions. Where possible these were conducted in locations to minimise distractions, however this was not always possible. Additionally, due to time restraints and a deliberate decision to not overburden patients while they were hospitalised we did not perform PHQ-9 and GAD-7 during hospitalisation. As such we could therefore only compare these at convalescence. While we endeavoured to recruit people of all ethnicities most of our study population was white which reflects the demographics of the local population. Finally, observational study designs preclude causal inference, though longitudinal data and biomarker correlations strengthen biological plausibility.

## 6 Conclusion

This exploratory study provides robust evidence for acute, inflammation-mediated brain injury in patients with BSI. Although cognition largely returned to normal at convalescence, mood disturbance persisted. Longitudinal study in larger cohorts and routine collection of cognitive and mood outcomes for patients with BSI, particularly in populations vulnerable to neurodegeneration, are justified to inform interventions aimed at mitigating chronic sequelae.

## Funding

This work was supported by the Medical Research Council [grant number MR/T023791/1] and the Hodge Centre for Translational Neuroscience.

## CRediT authorship contribution statement

**Jonathan Underwood:** Writing – review & editing, Writing – original draft, Project administration, Methodology, Investigation, Funding acquisition, Formal analysis, Data curation, Conceptualization. **Kate Davies:** Writing – review & editing, Investigation, Data curation. **Sam Loveless:** Writing – review & editing, Investigation, Data curation. **Mara Cercignani:** Writing – review & editing, Supervision, Software, Methodology, Formal analysis. **Nicholas G. Dowell:** Writing – review & editing, Software, Methodology, Formal analysis. **Eva Periche-Tomas:** Writing – review & editing, Methodology, Formal analysis. **Itamar Ronen:** Writing – review & editing, Software, Methodology, Formal analysis. **John Evans:** Writing – review & editing, Resources, Methodology, Data curation. **James E. McLaren:** Writing – review & editing, Supervision, Methodology, Data curation. **Neil A. Harrison:** Writing – review & editing, Writing – original draft, Methodology, Funding acquisition, Conceptualization.

## Declaration of Competing Interest

The authors declare that they have no known competing financial interests or personal relationships that could have appeared to influence the work reported in this paper.

## Data Availability

Participant-level data are not publicly available due to confidentiality and consent/ethics restrictions. Controlled access may be granted to bona fide researchers subject to Sponsor (Cardiff and Vale UHB) approval and a Data Sharing/Data Transfer Agreement. Requests via research.governance@wales.nhs.uk quoting IRAS project ID: 277598.
