# Supplementary material for: Bloodstream infections and Blood-Brain barrier Permeability: An observational cohort study
Source: Brain Behav Immun. 2026 Jul;135:None. doi: 10.1016/j.bbi.2026.106518 (PMC13365024; doi:10.1016/j.bbi.2026.106518)
Supplement: Supplementary Data 1 [file mmc1.docx]

# Supplementary data

## Supplementary Figure 1. Study overview


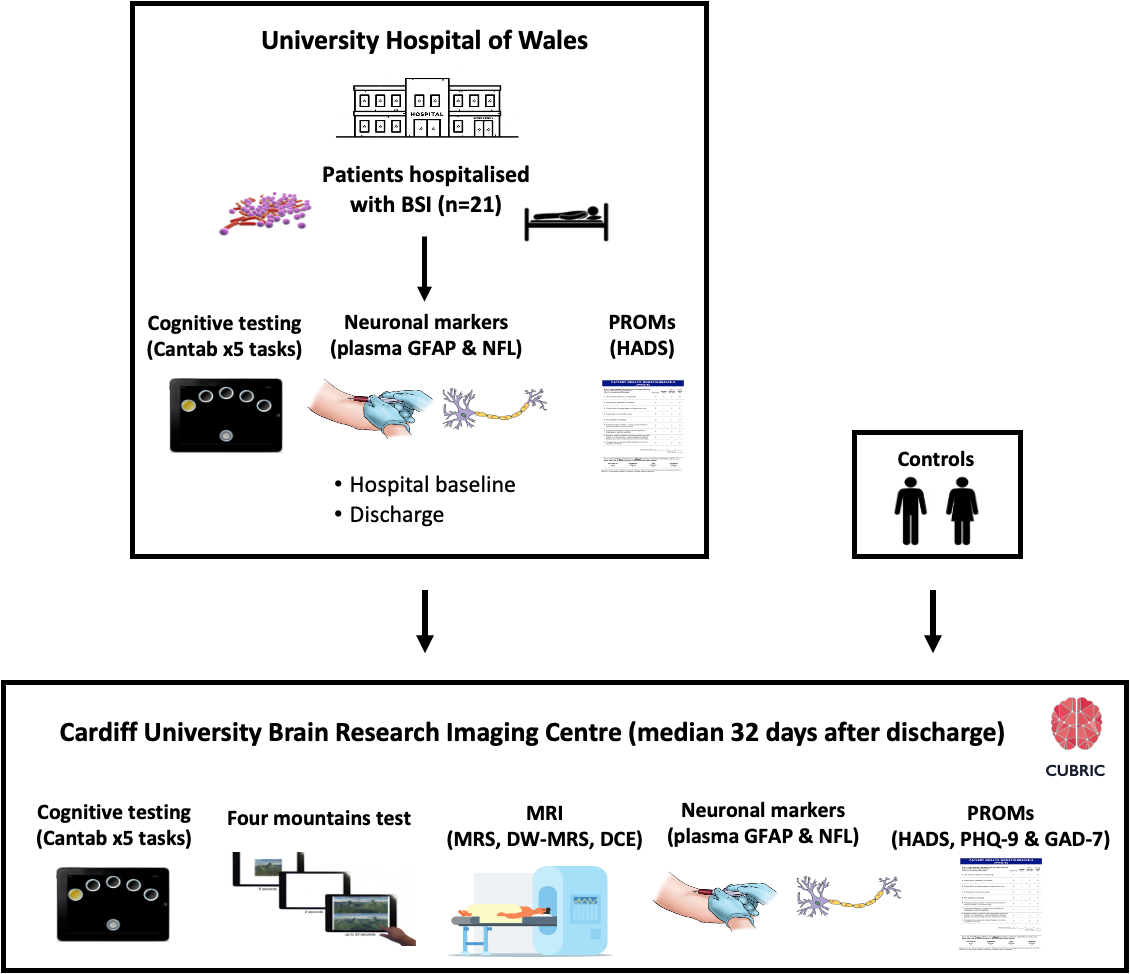


HADS: Hospital anxiety and depression score

NFL: neurofilament light protein

GFAP: glial fibrillary acidic protein

PHQ-9: Patient Health Questionnaire 9

GAD-7: General Anxiety Disorder 7

Supplementary figure 2. Example modelled Ktrans for a patient with BSI, overlaid on structural T1 image, with example segmentation, with acquired and modelled data.

##
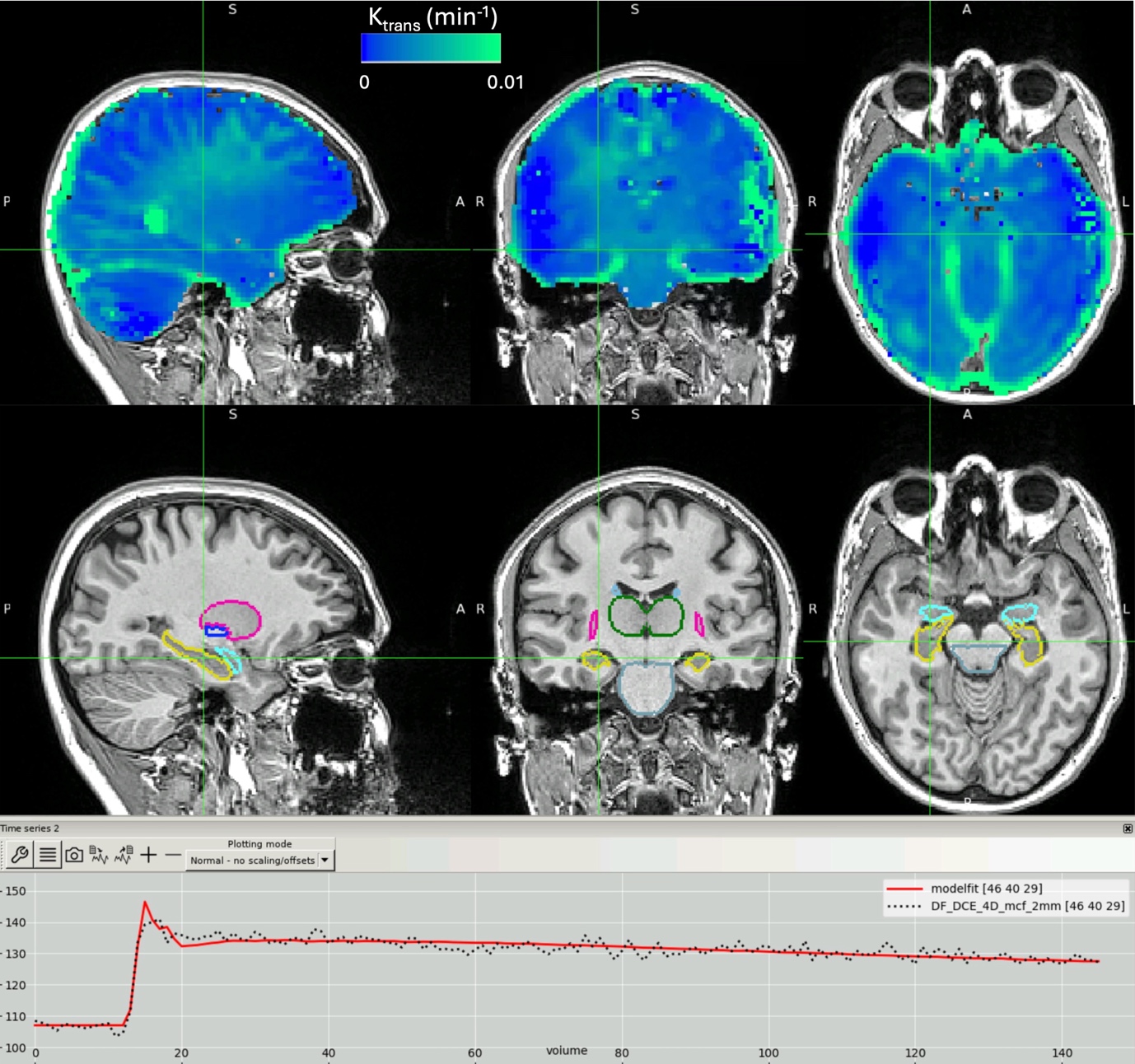


## Supplementary Figure 3. Patients with sepsis have poorer concentration and longer reaction times

**a**


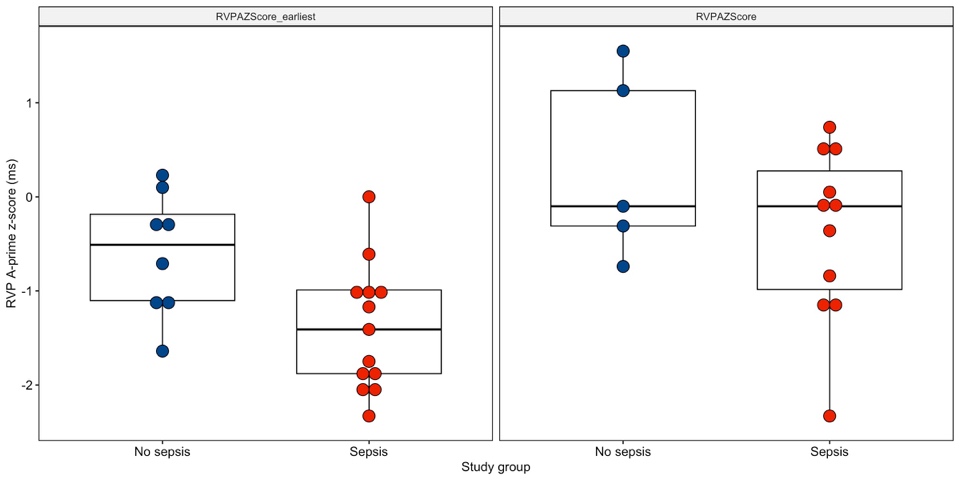


**b**


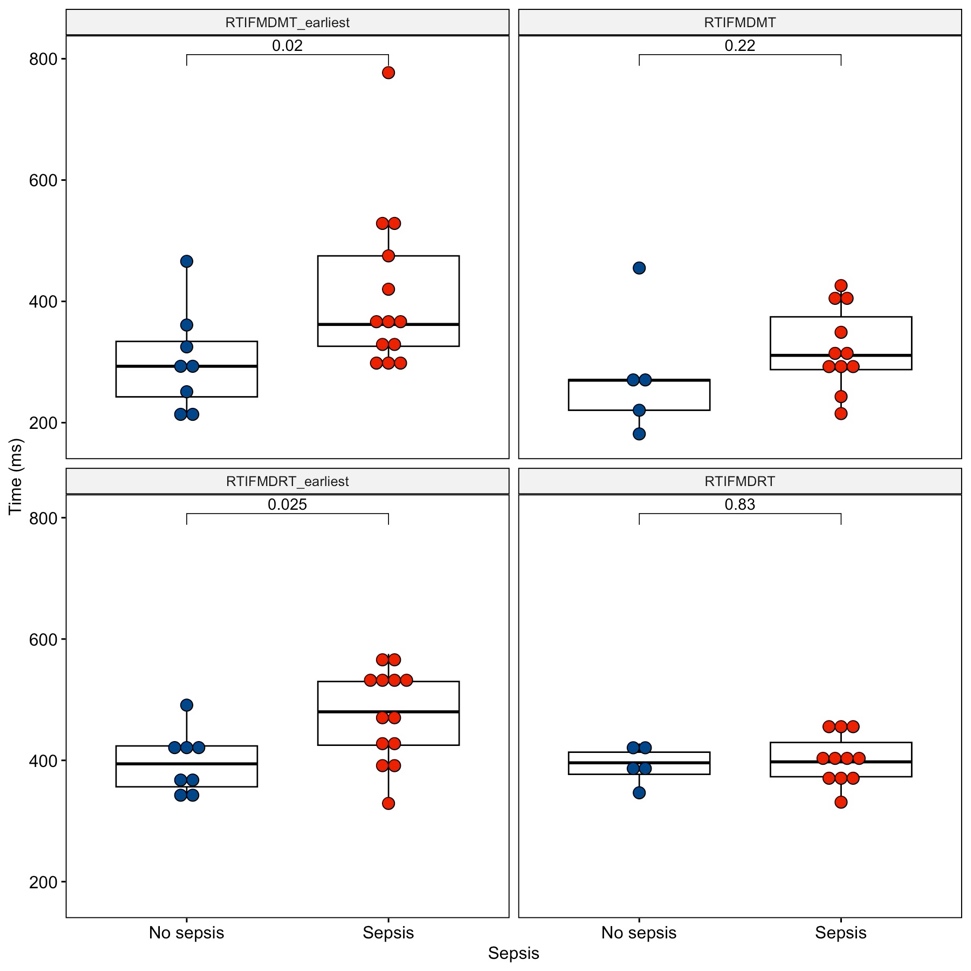


Patients with sepsis (defined as a sequential organ failure assessment – SOFA score of two or greater) had poorer attention (a) and longer reaction times (b) at hospital baseline (left panels) that normalised by convalescence (right panels).

RTI: choice reaction time task

RVP: rapid visual information processing task

## Supplementary Figure 4. Relationships between depression and anxiety scores and acute illness severity and systemic inflammation (BSI group only).

a


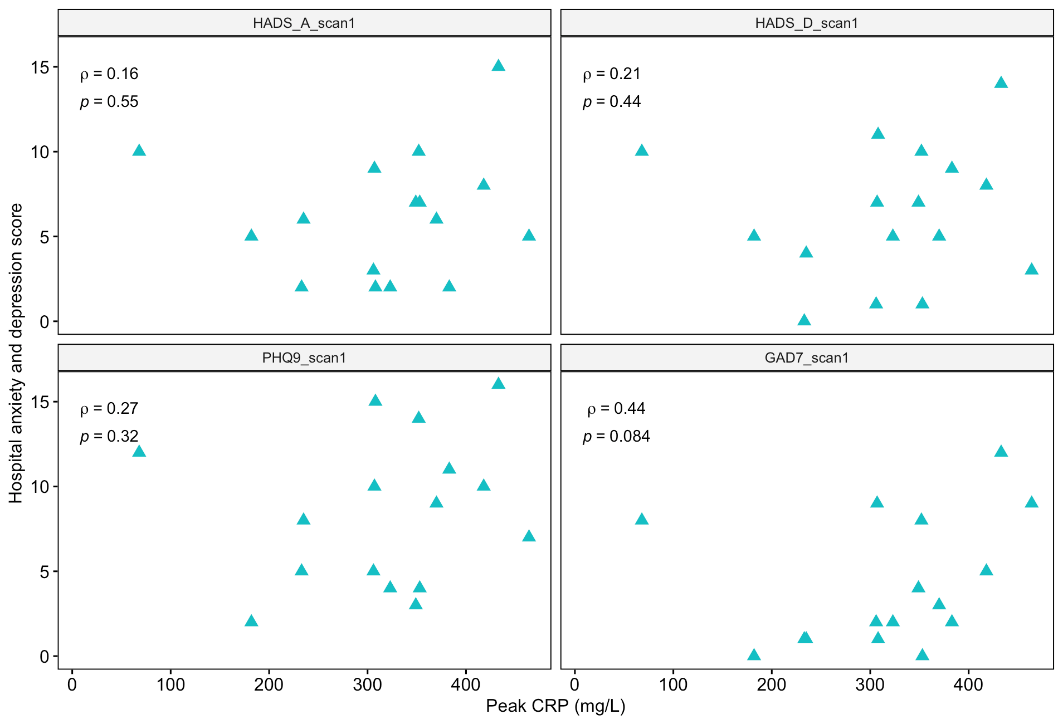


b


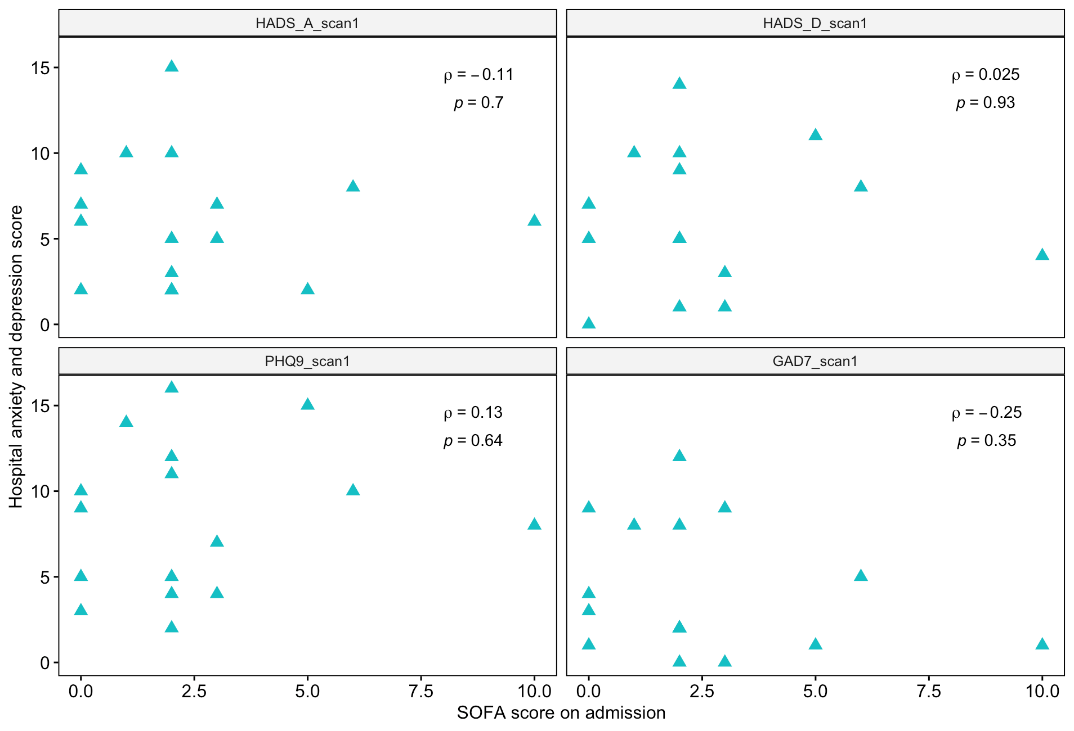


Scatterplots showing the relationship between the magnitude of the inflammatory response to bloodstream infection quantified by peak c-reactive protein (a) and severity of acute illness with symptoms of depression and anxiety.

HADS: Hospital anxiety and depression score

SOFA: sequential organ failure assessment

## Supplementary Figure 5. Axonal injury marker NFL is associated with severity of acute admission (BSI group only)


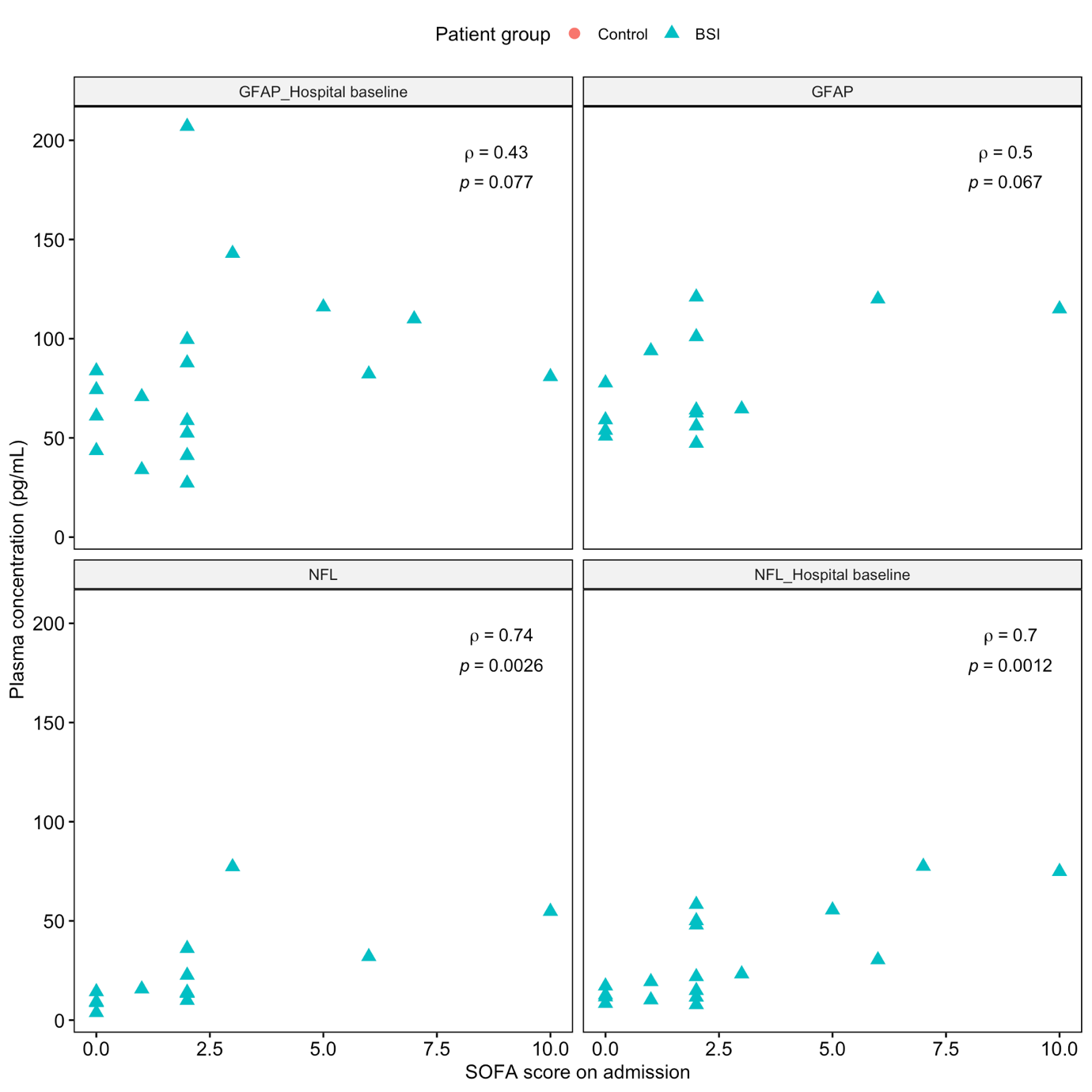


Scatterplots showing relationship between illness severity in patients with bloodstream infection, quantified with sequential organ failure assessment (SOFA) score, with markers of brain injury.

NFL: neurofilament light protein

GFAP: glial fibrillary acidic protein

## Supplementary Figure 6. Axonal injury marker NFL is associated with poorer cognitive function across multiple domains

##
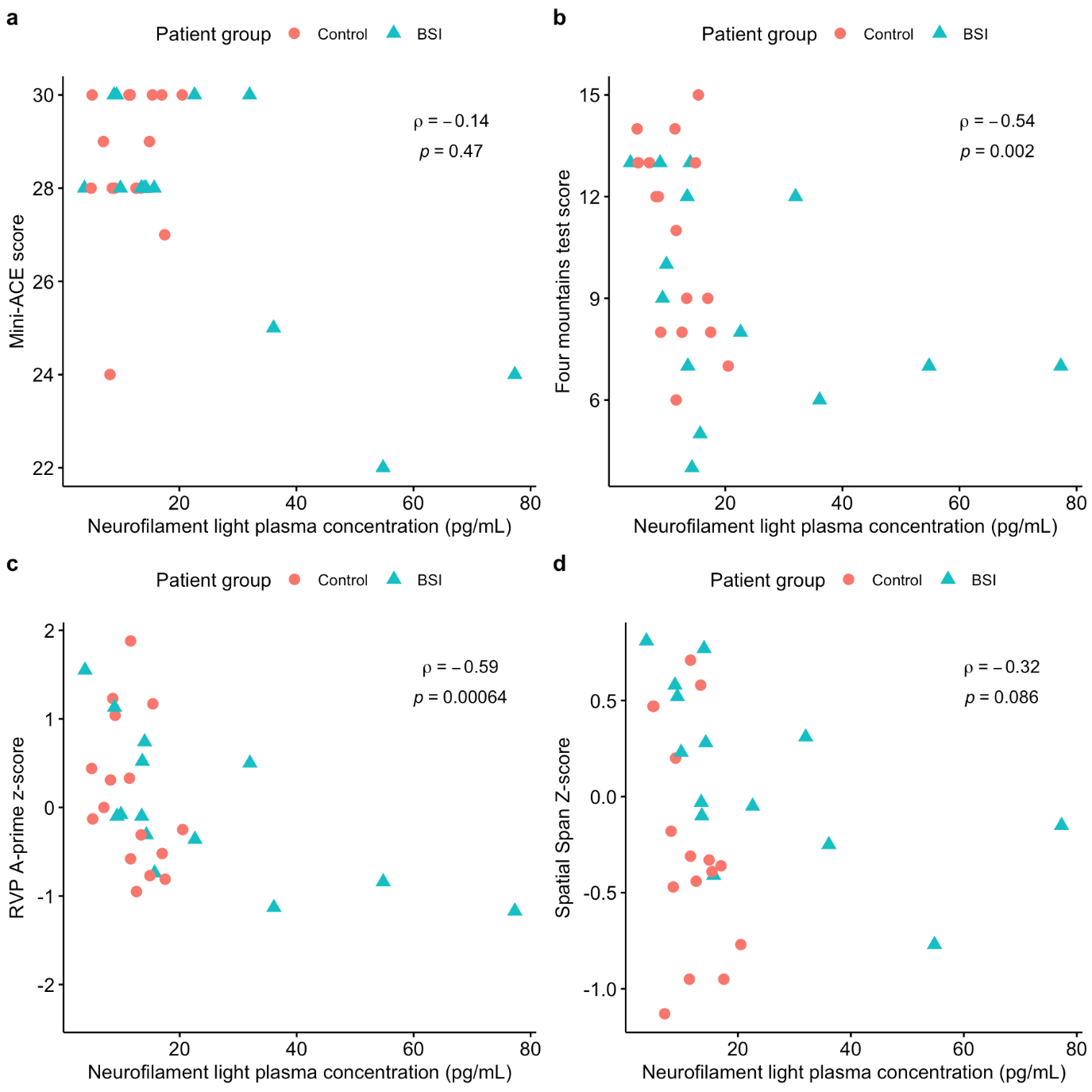


Scatterplots showing relationships between neurofilament light protein (NFL) and measures of cognitive function.

Mini-ACE: mini Addenbrooke’s cognitive examination

RVP: rapid visual information processing task

## Supplementary Figure 7. No difference in grey matter thickness or subcortical volumes at convalescence.

a


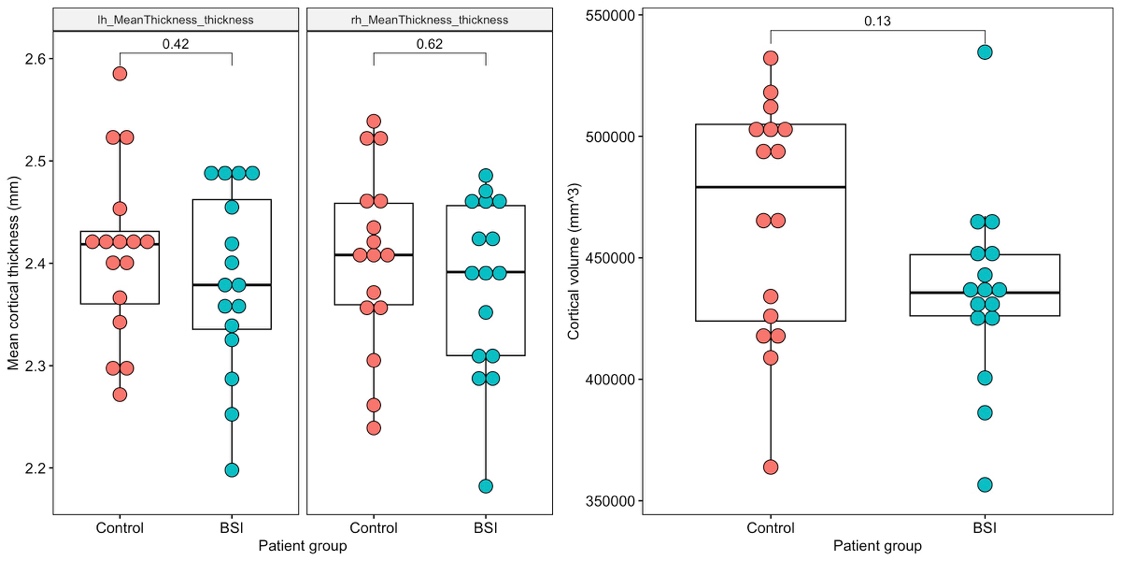


b


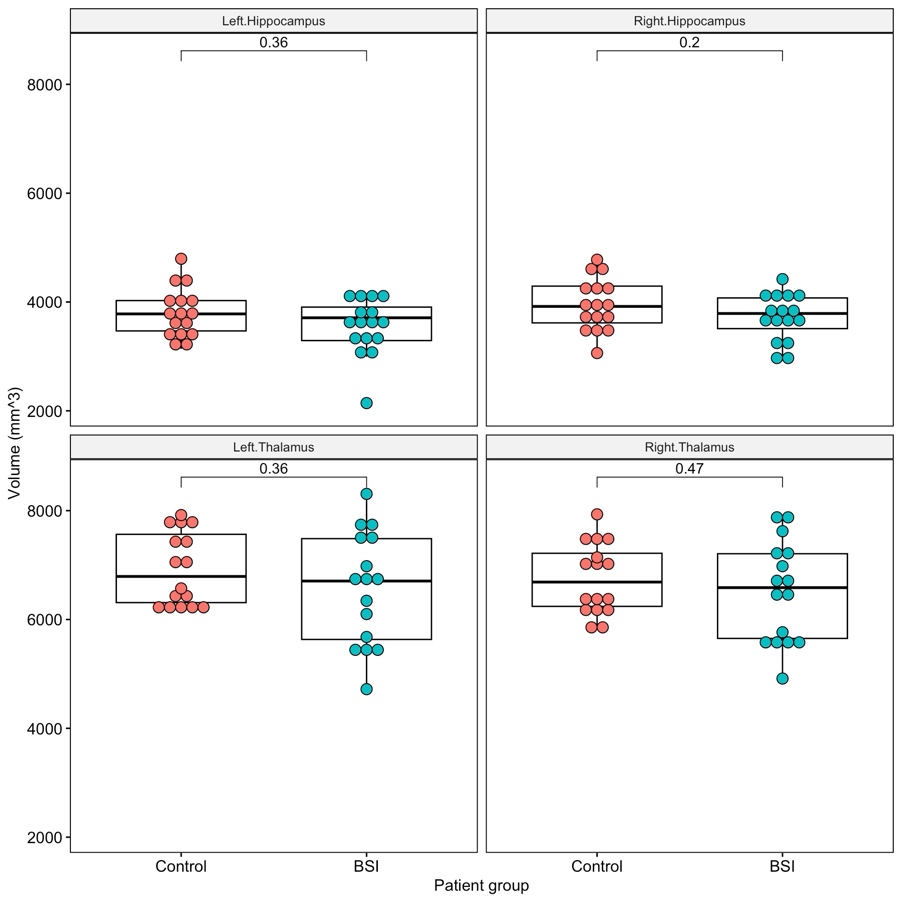


Boxplots of cortical thickness and cortical volume (a) and subcortical volumes (b) by patient group.

## Supplementary Figure 8. Increased grey matter and hippocampal BBB permeability is associated with poorer cognitive function across multiple domains.


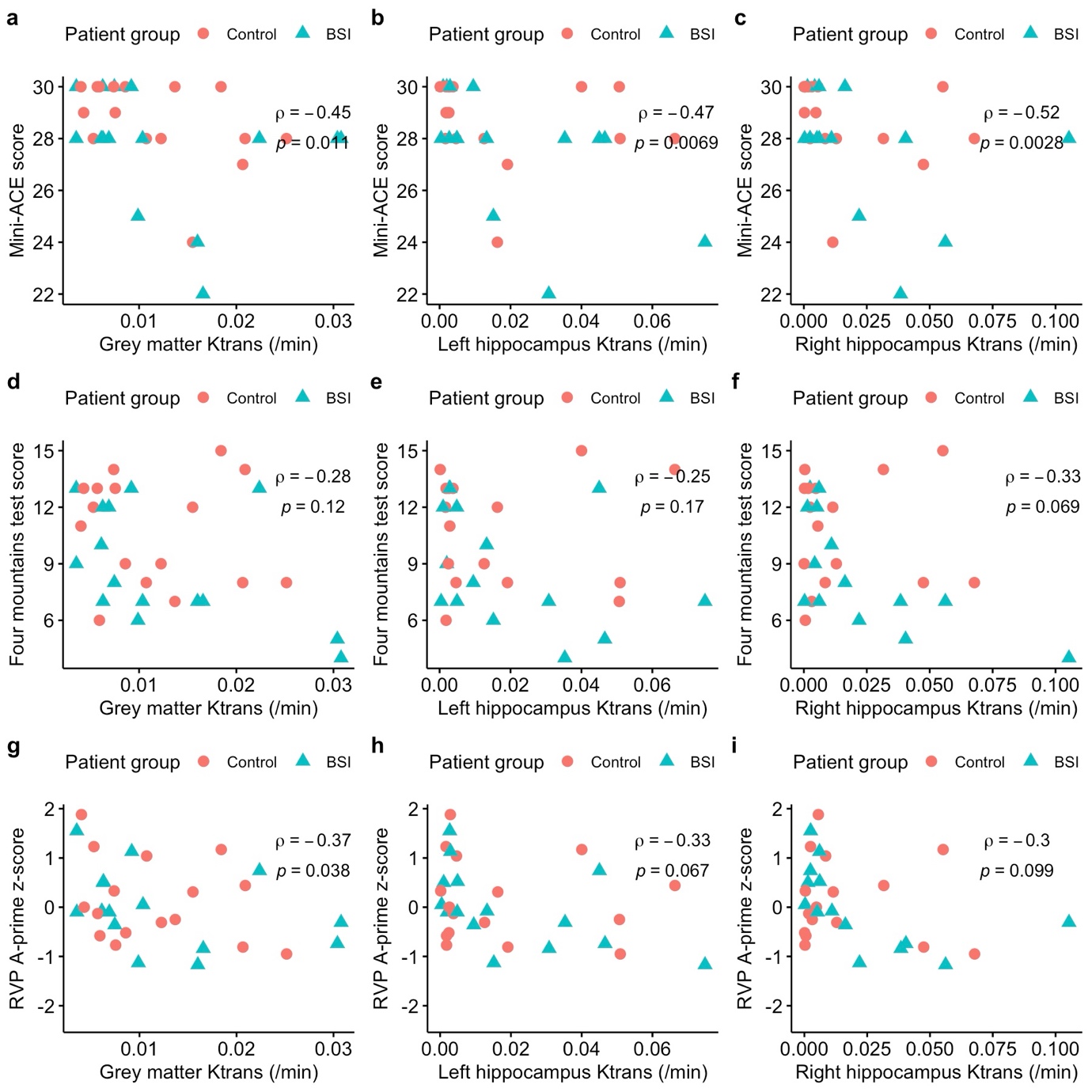


Scatterplots showing the relationship between blood-brain barrier permeability, quantified with using dynamic contrast enhanced MRI measure K_trans_, with measures of cognitive function.

Mini-ACE: mini Addenbrooke’s cognitive examination

RVP: rapid visual information processing task

## Supplementary Figure 9. Increased plasma NFL is associated with increased BBB permeability


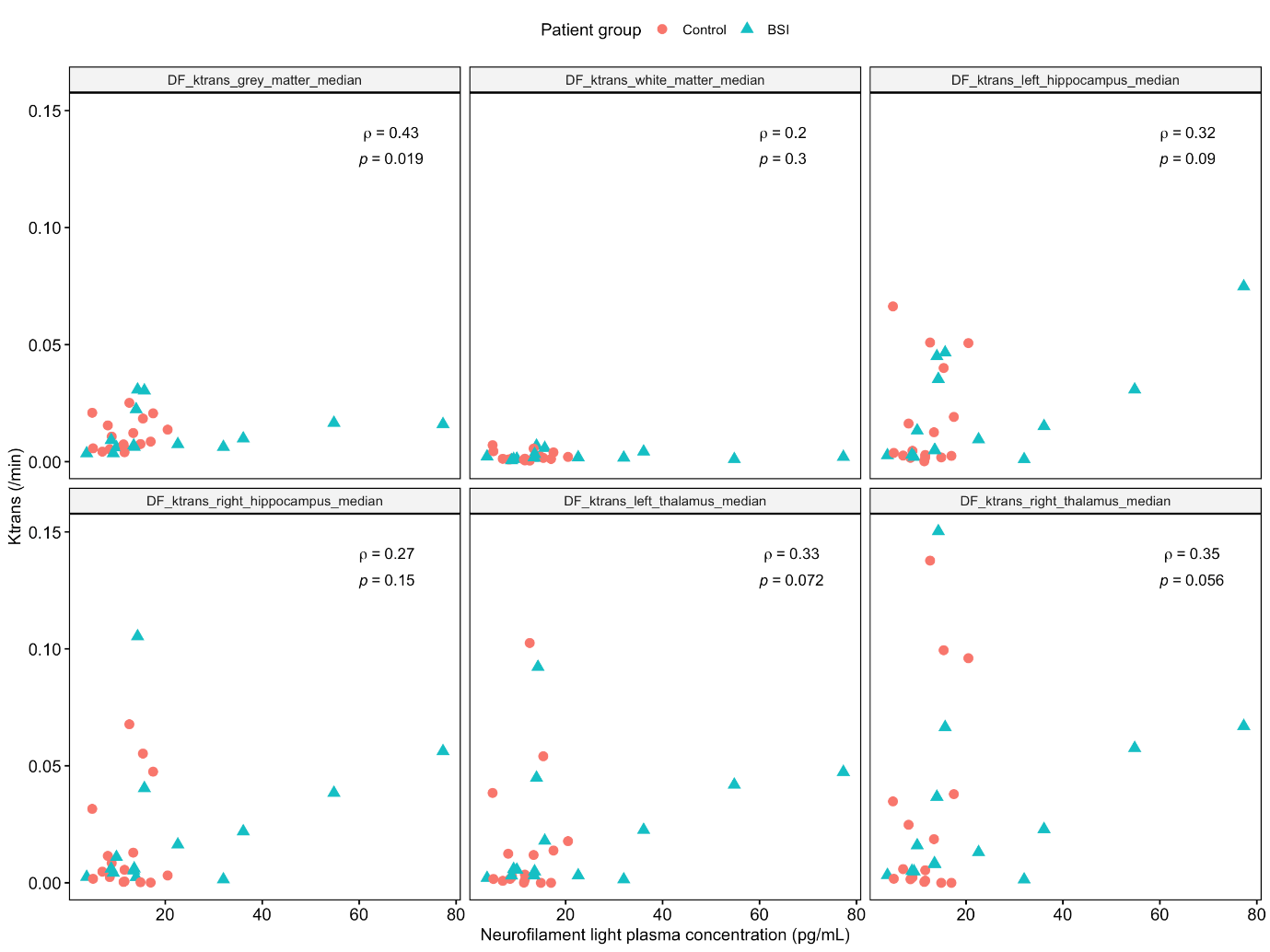


Scatterplots showing the relationship between plasma neurofilament light protein (NFL) with blood-brain barrier permeability, quantified with using dynamic contrast enhanced MRI measure K_trans_, across multiple brain regions.

## Supplementary Figure 10. No difference in absolute metabolite concentrations or apparent diffusion coefficients at convalescence

a


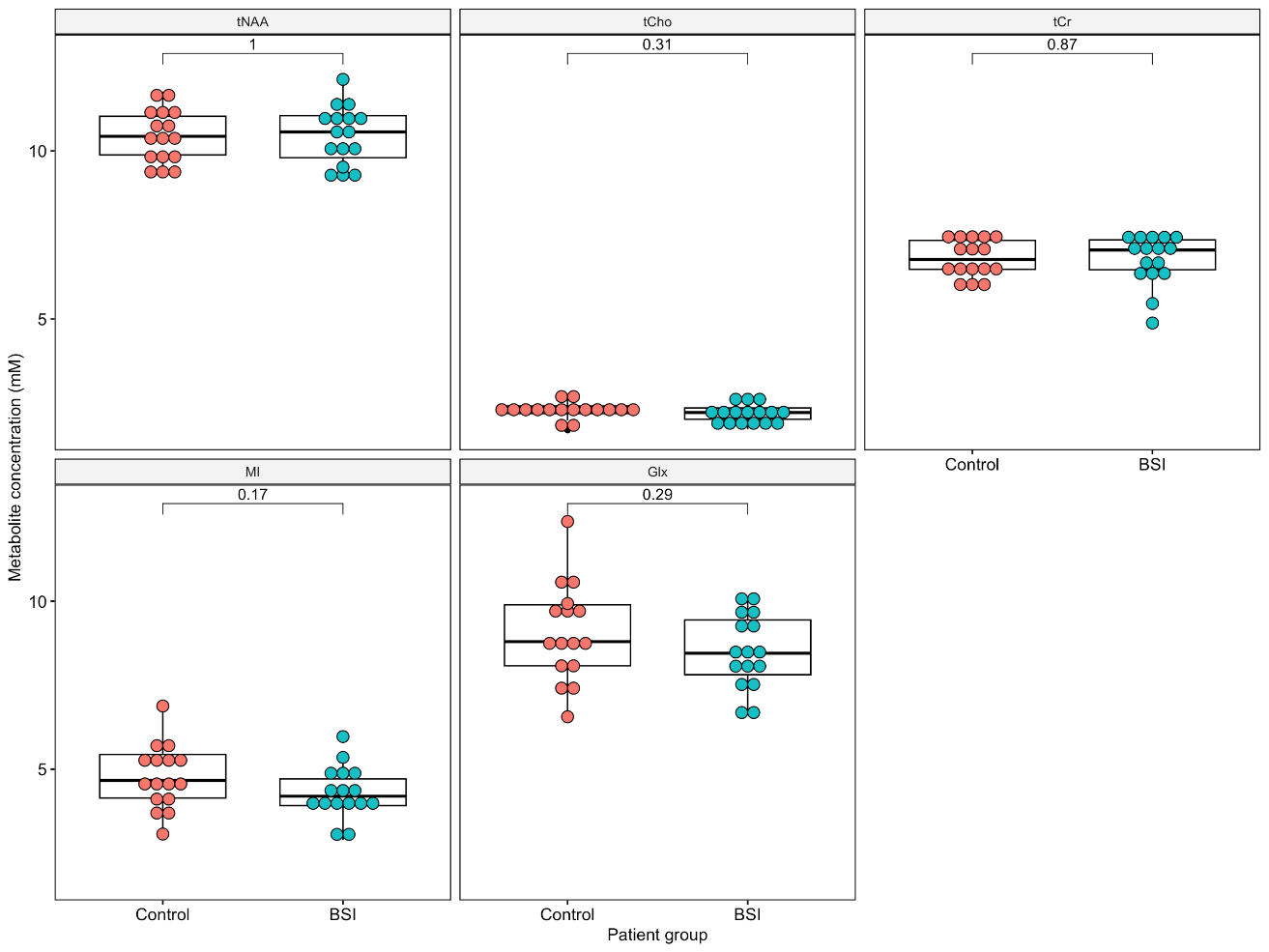


b


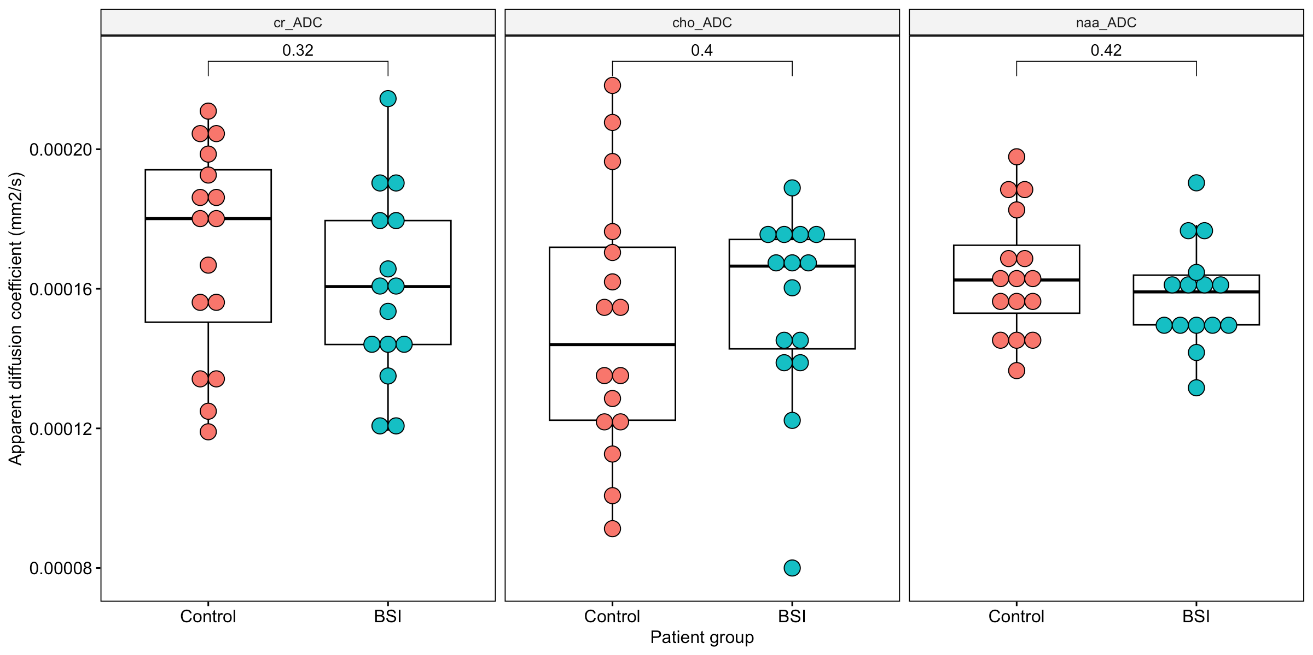


Boxplots of left thalamus metabolite absolute concentrations (a) and diffusion coefficients (b) by patient group.

Cho: choline; Cr: creatine; Glx: glutamate and glutamine; MI: myo-inositol; NAA: N-acetyl-aspartate (NAA)
